# Isolation of Novel Bacteria Including Rarely Cultivated Phyla, *Acidobacteria* and *Verrucomicrobia*, from the Roots of Emergent Plants by Simple Culturing Method

**DOI:** 10.1264/jsme2.ME17027

**Published:** 2017-09-27

**Authors:** Yasuhiro Tanaka, Hiroaki Matsuzawa, Hideyuki Tamaki, Masahiro Tagawa, Tadashi Toyama, Yoichi Kamagata, Kazuhiro Mori

**Affiliations:** 1 Department of Environmental Sciences, Faculty of Life and Environmental Sciences, University of Yamanashi 4–4–37 Takeda, Kofu, Yamanashi 400–8510 Japan; 2 International Research Centre for River Basin Environment, University of Yamanashi 4–3–11 Takeda, Kofu, Yamanashi 400–8511 Japan; 3 Bioproduction Research Institute, AIST 1–1–1 Higashi, Tsukuba, Ibaraki 305–8566 Japan; 4 Department of Civil and Environmental Engineering, Faculty of Engineering, University of Yamanashi 4–3–11 Takeda, Kofu, Yamanashi 400–8511 Japan

**Keywords:** emergent plant, *Acidobacteria*, *Verrucomicrobia*, microbial community

## Abstract

A number of novel bacteria including members of rarely cultivated phyla, *Acidobacteria* and *Verrucomicrobia*, were successfully isolated from the roots of two emergent plants, *Iris pseudacorus* and *Scirpus juncoides*, by a simple culturing method. A total of 47.1% (66 strains) for *I. pseudacorus* and 42.1% (59 strains) for *S. juncoides* of all isolates (140 strains from each sample) were phylogenetically novel. Furthermore, *Acidobacteria* and *Verrucomicrobia* occupied 10.7% (15 strains) and 2.9% (4 strains) of *I. pseudacorus* isolates, and 2.1% (3 strains) and 3.6% (5 strains) of *S. juncoides* isolates, respectively, indicating that plant roots are attractive sources for isolating rarely cultivated microbes.

Molecular ecological techniques based on 16S rRNA gene sequences have revealed that most prokaryotes in nature have yet to be cultured. The number of bacterial phyla was previously presumed to be 100, only 30 of which contain cultivated representatives according to the Greengenes and SILVA databases as of August 2014 (26). Hence, limited information is available on the entity of organisms in more than 70 phyla (candidate bacterial phyla). Furthermore, most of the cultivated microbes are affiliated with four bacterial phyla: *Actinobacteria*, *Bacteroidetes*, *Firmicutes*, and *Proteobacteria* (8), and another 26 bacterial phyla have a small number of axenic cultures and already reported species. The phyla *Acidobacteria* and *Verrucomicrobia* are widely distributed and abundantly detected in natural ecosystems (3–7, 11–13, 15, 16, 19, 25), but harbor only 37 and 43 authentic species, respectively, indicating that these groups mainly comprise rarely cultivated microbes. Many researchers have attempted to cultivate members of *Acidobacteria* and *Verrucomicrobia*, and, thus, novel microbes within these phyla were, to some extent, successfully isolated. However, extensive efforts and unique laborious strategies for cultivation are still needed, as follows: (i) *in situ* cultivation using diffusion chambers (1); and the combined use of (ii) a traditional plate culturing method or (iii) advanced cultivation techniques and screening methods with specific PCR primers and hybridization probes (10, 17, 18, 21).

Since there are very few studies on microbes associated with the roots of aquatic plants, we recently analyzed microbial communities in the roots of three aquatic plants: *Spirodela polyrhiza* (a floating aquatic plant), *Phragmites australis* (an emergent plant), and *Lythrum anceps* (an emergent plant), and succeeded in isolating a number of novel microbes including rarely cultivated organisms within the phyla *Verrucomicrobia* and *Armatimonadetes* (formerly called candidate phylum OP10) (14, 23). These findings prompted a tentative theory that aquatic plant roots may be an effective isolation source for a number of yet-to-be cultured microbes. In order to verify this hypothesis, further studies on microbial isolation targeting a greater variety of aquatic plant species are needed. In the present study, we conducted the isolation and phylogenetic characterization of microbes inhabiting the roots of two emergent plants, *Iris pseudacorus* and *Scirpus juncoides*, which are taxonomically far from (at the level of order) each other and the three already-targeted aquatic plants described above. We eventually found that root samples of the two aquatic plants were effective sources for isolating varieties of novel microbes representing members of rarely cultured bacterial phyla *Acidobacteria* and *Verrucomicrobia* by using a simple culturing method without an extensive effort.

Two plants, *I. pseudacorus* and *S. juncoides*, were harvested from a pond in Yamanashi prefectural wood park “Kanegawa-no-mori” (Fuefuki, Yamanashi, Japan; 35°38′23″ N, 138°40′36″ E). In order to remove free-living microbes in pond water, which are unlikely to interact with the roots of plants, root samples (0.3 g wet weight) were gently rinsed twice with 30 mL of sterilized modified Hoagland solution (24), and applied to total DNA extraction using the ISOIL kit for bead beating (Nippon gene, Tokyo, Japan). The Ultraclean water DNA kit (MO BIO Laboratories, Carlsbad, CA, USA) was used for DNA extraction from a pond water sample (100 mL) collected near the plants. The PCR amplification of 16S rRNA gene fragments was conducted using a previously described method (14) with two bacterial universal primers, Eub8F and Eub1512R, except for the number of cycles; these numbers were adjusted to 19, 20, and 21 cycles for *I. pseudacorus*, *S. juncoides*, and pond water, respectively, in order to reduce PCR bias. Amplified DNA fragments were purified using the illustra GFX PCR purification kit (GE Healthcare Life Sciences, Pittsburgh, PA, USA), ligated into the pT7 Blue T-vector (Takara, Otsu, Japan), and the resultant recombinant plasmids were transformed into *E. coli* strain DH5α-competent cells (Takara).

The clonal 16S rRNA fragment was amplified and subjected to a restriction fragment length polymorphism (RFLP) analysis by separate digestion with *Hha*I and *Hae*III (Takara), as reported in our previous study (14). Coverage (*C*) values for each clone library were calculated by the equation *C*=[1–(*n/N*)]×100, where *n* is the number of unique clones and *N* is the total number of clones analyzed.

Low-nutrient media, DTS (pH 7.0) medium (14) and DR2 medium (22), solidified with two types of gelling reagents, 1.5% agar or 1.0% gellan gum, were used for the isolation of microbes in order to capture a wider variety of microbes, as reported by Tamaki *et al.* (22).

Approximately 0.2 g (wet weight) of root samples were washed for a molecular-based microbial analysis, and the washed samples were mechanically homogenized as described in our previous study (23). The homogenate or pond water sample was diluted in 10-fold steps using modified Hoagland solution, and 50 μL of each dilution was independently spread onto plates and incubated at 25°C for 37 d under dark conditions.

The 16S rRNA genes of the isolates were amplified by colony direct PCR using Eub8F and Eub1512R primers, and subjected to a RFLP analysis using similar methods to those for a molecular-based microbial community analysis with the exception that *Msp*I (Takara) was used instead of *Hae*III.

The amplified 16S rRNA gene fragments of representative clones or isolates of each RFLP phylotype were purified with the GFX PCR DNA and Gel purification kit (GE Healthcare), after which they were sequenced as described in our previous study (14). The data obtained were compared with those in EZ BioCloud (http://www.ezbiocloud.net/). The taxonomic classification of the sequence at the class level was conducted using the CLASSIFIER program (http://rdp.cme.msu.edu/classifier/classifier.jsp).

In order to verify whether the two aquatic plants used in this study harbor abundant yet-to-be cultured microbes in their roots, the microbial communities in the roots were analyzed with a culture-independent method based on the 16S rRNA gene sequence. As a comparative experiment, we also analyzed the microbial community in pond water collected near the plants. Ninety-five clones from each 16S rRNA gene clone library were subjected to a RFLP analysis, which divided the clones from *I. pseudacorus*, *S. juncoides*, and pond water libraries into 69, 77, and 59 phylotypes, respectively (Table S1). Coverage values were 29% for *I. pseudacorus*, 32% for *S. juncoides*, and 54% for pond water. Three diversity indices: the Shannon-Weiner index, Simpson’s reciprocal index, and Probability of Interspecific Encounter (PIE) index (9), based on the grouping of phylotypes revealed that the microbial communities of two plants were more diverse than that of pond water (Table 1).

In the phylogenetic analysis based on the representative clone sequence of each phylotype, 13 bacterial phyla were detected, and 9, 9, and 7 of them were found in the clone libraries of *I. pseudacorus*, *S. juncoides*, and pond water, respectively (Table 2, Fig. S1). Although *Proteobacteria* was the predominant bacterial phylum in the two plant samples with the same abundance (56.8%), the bacterial composition ratios at the class level within the phylum were different (Table 2). Rarely cultivated microbial groups, *Acidobacteria*, *Armatimonadetes*, *Chloroflexi*, *Planctomycetes*, and *Verrucomicrobia*, were also detected, and the clones affiliated with these phyla, candidate phylum, and unclassifiable group accounted for 19.0% of all clones from *I. pseudacorus* and 29.6% from *S. juncoides*, while the score for pond water was very low (4.3%) (Table 2, Fig. S2). In addition, when clonal sequences indicating less than 97% 16S rRNA gene similarity to any known bacterial species were regarded as phylogenetically novel microbes, as defined by Stackebrandt *et al.* (20), 68.4% and 73.7% of the clones derived from *I. pseudacorus* and *S. juncoides*, respectively, cleared this criterion, whereas the score of pond water was 45.3% (Fig. S2). Aquatic plant root-inhabiting microbes must originally be from pond water. Therefore, these differences in microbial diversity and novelty between plant root and pond water samples are of interest. There are several plausible reasons for the differences observed: 1) the abundance of the novel microbes including rarely cultivated phyla in the pond water sample exceeded the lower limit of detection by the clone library analysis, 2) the microbial consortium in pond water continually fluctuates, and, thus, the microbes specifically detected in plant samples were not present in the water sample examined in the present study, 3) aquatic plants select their favorite microbes in their roots, which may be taxonomically novel, from the microbial consortia in pond water, and then enhance the cell growth and activity of root-inhabiting microbes. In any case, the results shown above indicated that the aquatic plants used in the present study harbored plant-specific and various microbes including abundant yet-to-be cultured bacteria in their roots, and strongly suggest that these features are ubiquitously in emergent plants because the same findings were obtained in our previous study targeting other emergent plants, *L. anceps* and *P. australis* (23).

The cultivation and isolation of novel microbes inhabiting the roots of *I. pseudacorus* and *S. juncoides* were conducted. Regarding cultivation, a simple plate culturing method using four types of media: agar-based DTS (DTSA), gellan gum-based DTS (DTSG), agar-based DR2 (DR2A), and gellan gum-based DR2 (DR2G) plates, was employed. A comparative test targeting pond water collected from near the plants was also conducted using DTS-derived media. After cultivation, forty-five colonies from each DTS-derived plate and 25 colonies from each DR2-derived plate were randomly selected. The 16S rRNA gene sequences of the isolates (370 isolates) were divided into 144 phylotypes by a RFLP analysis (Table S2). The scores of the Shannon-Weiner, Simpson’s reciprocal, and PIE indices based on the RFLP phylotypes from DTS-derived plates indicated that isolates from plants were more diverse than those from pond water, as shown in the molecular-based microbial community analysis (Table 1). The PIE index, which is known to be unbiased by sampling size in contrast to the two other indices, was used to compare diversities among the isolates from four types of media in two aquatic plants, indicating that the varieties of isolates were not largely affected by differences in the plate medium used and plant species because all the scores were calculated within a relatively narrow range (0.923–0.974).

The 16S rRNA gene sequences of the representative isolates for each RFLP phylotype were elucidated, and then compared with those in the EZ BioCloud database. The most closely related species of the isolates are shown in Table S2. When the taxonomic novelty of the isolates was judged by the same criterion defined in the molecular-based analysis, 47.1% of all isolates (35.6% for DTSA, 53.3% for DTSG, 48.0% for DR2A, and 56.0% for DR2G isolates) from *I. pseudacorus*, and 42.1% of those (28.9% for DTSA, 44.4% for DTSG, 56.0% for DR2A, and 48.0% for DR2G isolates) from *S. juncoides* were novel microbes (Fig. S2). In contrast, the abundance of the novel microbes from pond water remained at 11.1% (6.7% and 15.6% in DTSA and DTSG, respectively). Phylogenetic classification at the phylum level indicated that the isolates were divided into five bacterial phyla consisting of three frequently cultivated bacterial phyla (*Proteobacteria*, *Actinobacteria*, and *Bacteroidetes*) and two rarely cultivated bacterial phyla (*Acidobacteria* and *Verrucomicrobia*) (Table 2, Fig. S1). Members of *Acidobacteria* and *Verrucomicrobia* were not obtained from pond water, they were only gained from aquatic plant samples. The abundance of these fastidious microbes belonging to *Acidobacteria* and *Verrucomicrobia* accounted for 13.6% (8.9% for DTSA, 15.5% for DTSG, 20.0% for DR2A, and 12.0% for DR2G isolates) and 5.7% (4.4% for DTSA, 11.1% for DTSG, 4.0% for DR2A, and 0% for DR2G isolates) of all isolates from *I. pseudacorus* and *S. juncoides*, respectively (Table 2).

Several approaches to cultivate microbes within the phyla *Acidobacteria* and *Verrucomicrobia* have been reported, most of which require a cumbersome step (*e.g.*, a screening procedure using a specific primer and/or probe targeting two groups) (17, 21). However, even a very simple cultivation method allowed us to isolate novel members of *Acidobacteria* and *Verrucomicrobia* from the roots of *I. pseudacorus* and *S. juncoides* in the present study. Plant root exudates may be one of the main factors for the successful cultivation of rarely cultivated members. Plant root exudates are known to affect microbial activity, metabolism, and colonization in the rhizosphere of terrestrial plants (2), and may function to wake up dormant or inactive microbes into readily cultivable ones. In addition, in the case of *Acidobacteria*, the high yield of their abundances in root samples (8.4% for *I. pseudacorus* and 18.9% for *S. juncoides* in culture-independent analysis) and the use of low-nutrient media, which often led to the isolation of fastidious microbes (22), may also contribute to successful isolation.

Taken together with our previous study, the present results support the hypothesis that the roots of aquatic plants are commonly useful sources for excavating novel microbes, and the types of cultivable microbes differ among the plant species. Therefore, further and more thorough microbial isolation attempts that target a wide variety of aquatic plants will be very effective and important from the point of view of developing untapped microbial and genetic resources even though it is slightly tedious work.

## Nucleotide sequence accession numbers

The GenBank/EMBL/DDBJ accession numbers for the 16S rRNA gene sequences of clones and isolates are LC209232–LC209429 and LC106157–LC106300, respectively.

## Supplementary Material



## Figures and Tables

**Table 1:** Diversity indices for clones and isolates

Diversity indices	Clones			Isolates									
	
	*Iris pseudacorus*	*Scirpus juncoides*	Pond water	*Iris pseudacorus*				*Scirpus juncoides*				Pond water	
		
				DTSA	DTSG	DR2A	DR2G	DTSA	DTSG	DR2A	DR2G	DTSA	DTSG
Richness*	69 (95)	77 (95)	59 (95)	27 (45)	28 (45)	18 (25)	19 (25)	26 (45)	26 (45)	20 (25)	12 (25)	10 (45)	12 (45)
Shannon-Weiner index	4.02	4.24	3.79	3.15	3.19	2.81	2.84	3.03	3.06	2.84	2.31	1.62	1.84
Simpson’s reciprocal index	39.4	58.2	29.8	20.0	20.9	15.2	15.2	15.9	17.3	13.3	8.80	3.34	4.23
PIE index	0.985	0.993	0.976	0.972	0.974	0.973	0.973	0.959	0.964	0.963	0.923	0.716	0.781

* Richness is the number of phylotypes of clones or isolates. The number of clones or isolates obtained in the present study was shown in parentheses.

**Table 2:** Taxonomic classification of clones and isolates

Phylum	Class	Number of clones			Number of isolates												
	
		*Iris pseudacorus*	*Scirpus juncoides*	Pond water	*Iris pseudacorus*					*Scirpus juncoides*					Pond water		
		
					DTSA	DTSG	DR2A	DR2G	Total	DTSA	DTSG	DR2A	DR2G	Total	DTSA	DTSG	Total
***Proteobacteria***		**54 (56.8)**	**54 (56.8)**	**57 (60.0)**	**39 (86.7)**	**36 (80.0)**	**18 (72.0)**	**20 (80.0)**	**113 (80.7)**	**42 (93.3)**	**37 (82.2)**	**24 (96.0)**	**25 (100)**	**128 (91.4)**	**42 (93.3)**	**21 (46.7)**	**63 (70.0)**
	*Alphaproteobacteria*	27 (28.4)	17 (17.9)	6 (6.3)	29 (64.4)	21 (46.7)	14 (56.0)	13 (52.0)	77 (55.0)	16 (35.6)	18 (40.0)	15 (60.0)	15 (60.0)	64 (45.7)	9 (20.0)	16 (35.6)	25 (27.8)
	*Betaproteobacteria*	21 (22.1)	27 (28.4)	44 (46.3)	10 (22.2)	15 (33.3)	4 (16.0)	7 (28.0)	36 (25.7)	26 (57.8)	19 (42.2)	8 (32.0)	10 (40.0)	63 (45.0)	33 (73.3)	1 (2.2)	34 (37.8)
	*Gammaproteobacteria*		4 (4.2)	4 (4.2)								1 (4.0)		1 (0.7)		4 (8.9)	4 (4.4)
	*Deltaproteobacteria*	6 (6.3)	6 (6.3)	2 (2.1)													
	Unclassified			1 (1.1)													

***Acidobacteria***		**8 (8.4)**	**18 (18.9)**		**3 (6.7)**	**5 (11.1)**	**4 (16.0)**	**3 (12.0)**	**15 (10.7)**	**1 (2.2)**	**2 (4.4)**			**3 (2.1)**			
	*Holophagae*	2 (2.1)					1 (4.0)		1 (0.7)								
	*Acidobacteriia*	5 (5.3)			3 (6.7)	5 (11.1)	2 (8.0)	3 (12.0)	13 (9.3)	1 (2.2)	2 (4.4)			3 (2.1)			
	*Solibacteres*		2 (2.1)														
	Subdivision 3	1 (1.1)	15 (15.8)				1 (4.0)		1 (0.7)								
	Subdivision 6		1 (1.1)														

***Actinobacteria***		**2 (2.1)**	**4 (4.2)**	**7 (7.4)**	**1 (2.2)**	**1 (2.2)**	**2 (8.0)**	**1 (4.0)**	**5 (3.6)**	**1 (2.2)**	**2 (4.4)**			**3 (2.1)**	**2 (4.4)**		**2 (2.2)**
	*Actinobacteria*	2 (2.1)	4 (4.2)	7 (7.4)	1 (2.2)	1 (2.2)	2 (8.0)	1 (4.0)	5 (3.6)	1 (2.2)	2 (4.4)			3 (2.1)	2 (4.4)		2 (2.2)

***Armatimonadetes***		**2 (2.1)**	**1 (1.1)**	**1 (1.1)**													
	*Armatimonadia*		1 (1.1)														
	Unclassified	2 (2.1)		1 (1.1)													

***Bacteroidetes***		**11 (11.6)**	**3 (3.2)**	**23 (24.2)**	**1 (2.2)**	**1 (2.2)**		**1 (4.0)**	**3 (2.1)**		**1 (2.2)**			**1 (0.7)**	**1 (2.2)**	**24 (53.3)**	**25 (27.8)**
	*Bacteroidia*	1 (1.1)															
	*Cytophagia*			6 (6.3)													
	*Flavobacteriia*			5 (5.3)											1 (2.2)	23 (51.1)	24 (26.7)
	*Sphingobacteriia*	1 (1.1)	3 (3.2)	9 (9.5)	1 (2.2)	1 (2.2)			2 (1.4)		1 (2.2)			1 (0.7)		1 (2.2)	1 (1.1)
	Unclassified	9 (9.5)		3 (3.2)				1 (4.0)	1 (0.7)								

***Chloroflexi***		**1 (1.1)**															
	Unclassified	1 (1.1)															

***Cyanobacteria***			**6 (6.3)**	**2 (2.1)**													
	*Cyanobacteria*		6 (6.3)	2 (2.1)													

***Firmicutes***		**6 (6.3)**															
	*Clostridia*	3 (3.2)															
	*Negativicutes*	2 (2.1)															
	Unclassified	1 (1.1)															

***Fusobacteria***				**2 (2.1)**													
	*Fusobacteriia*			2 (2.1)													

**“Latescibacteria”**			1 (1.1)														
	Unclassified		1 (1.1)														

***Planctomycetes***			**3 (3.2)**														
	*Planctomycetacia*		3 (3.2)														

***Spirochaetes***		**4 (4.2)**															
	*Spirochaetes*	4 (4.2)															

***Verrucomicrobia***		**4 (4.2)**	**2 (2.1)**	**2 (2.1)**	**1 (2.2)**	**2 (4.4)**	**1 (4.0)**		**4 (2.9)**	**1 (2.2)**	**3 (6.7)**	**1 (4.0)**		**5 (3.6)**			
	*Opitutae*			2 (2.1)	1 (2.2)	2 (4.4)	1 (4.0)		4 (2.9)	1 (2.2)	3 (6.7)	1 (4.0)		5 (3.6)			
	*Spartobacteria*		1 (1.1)														
	*Verrucomicrobiae*	1 (1.1)															
	Subdivision 3	3 (3.2)	1 (1.1)														

**Unclassified**		**3 (3.2)**	**3 (3.2)**	**1 (1.1)**													

	Total	95 (100)	95 (100)	95 (100)	45 (100)	45 (100)	25 (100)	25 (100)	140 (100)	45 (100)	45 (100)	25 (100)	25 (100)	140 (100)	45 (100)	45 (100)	90 (100)

Abundances (%) of clones and isolates were shown in parentheses. Bold type indicates the numbers and abundances of microbes at the phylum level.
