# Supplementary material for: Isolation of Novel Bacteria Including Rarely Cultivated Phyla, Acidobacteria and Verrucomicrobia, from the Roots of Emergent Plants by Simple Culturing Method
Source: Microbes Environ. 2017 Sep 27;32(3):288–92. doi: 10.1264/jsme2.ME17027 (PMC5606700; doi:10.1264/jsme2.ME17027)
Supplement: Supplementary file 1 [file 32_288_s1.pdf]

Table S1. Phylogenetic affiliations of 16S rRNA gene clones

Clone-phylotype No.	Total no. of clones			Related authentic species			Phylum (Class) classified by CLASSIFIER	Compared length (bp)
	Iris pseudacorus	*Scirpus juncoides*	Pond water	Specis (Accession no.)	Phylum (Class)	Identity (%)		
1	1			*Geothrix fermentans* (U41563)	*Acidobacteria*	96.71	● *Acidobacteria*	790
2	1			*Methylocystis heyeri* (AM283543)	*Proteobacteria* (Alpha)	94.29	● *Proteobacteria* (Alpha)	805
3	1			*Methylocystis heyer* (AM283543)	*Proteobacteria* (Alpha)	93.93	● *Proteobacteria* (Alpha)	758
4	1			*Methylocystis heyeri* (AM283543)	*Proteobacteria* (Alpha)	94.06	● *Proteobacteria* (Alpha)	758
5	1			*Novosphingobium lentum* (AJ303009)	*Proteobacteria* (Alpha)	97.69	● *Proteobacteria* (Alpha)	735
6	2			*Pelobacter propionicus* (X70954)	*Proteobacteria* (Delta)	98.01	● *Proteobacteria* (Delta)	722
7	1			*Pelobacter propionicus* (X70954)	*Proteobacteria* (Delta)	96.42	● *Proteobacteria* (Delta)	755
8	1			*Novosphingobium hassiacum* (AJ416411)	*Proteobacteria* (Alpha)	97.86	● *Proteobacteria* (Alpha)	702
9	4			*Rhodomicrobium vannielii* (FN666247)	*Proteobacteria* (Alpha)	97.28	● *Proteobacteria* (Alpha)	773
10	1			*Dehalogenimonas alkenigignens* (JQ994266)	*Chloroflexi*	85.10	● *Chloroflexi*	719
11	1			*Clostridium saccharoperbutylacetonicum* (U16122)	*Firmicutes*	99.45	● *Firmicutes*	729
12	1			*Clostridium saccharoperbutylacetonicum* (U16122)	*Firmicutes*	99.56	● *Firmicutes*	687
13	1			*Fimbriimonas ginsengisoli* (GQ339893)	*Armatimonadetes*	88.32	● *Armatimonadetes*	737
14	1			*Clostridium saccharoperbutylacetonicum* (U16122)	*Firmicutes*	99.87	● *Firmicutes*	756
15	1			*Leptospira terpstrae* (AOGW02000008)	*Spirochaetes*	83.86	● Unclassified	591
16	3			*Rhodoblastus acidophilus* (FR733696)	*Proteobacteria* (Alpha)	98.84	● *Proteobacteria* (Alpha)	777
17	1			*Paludibacter propionicigenes* (AB078842)	*Bacteroidetes*	88.52	● *Bacteroidetes*	775
18	1			*Rhodoblastus acidophilus* (FR733696)	*Proteobacteria* (Alpha)	98.71	● *Proteobacteria* (Alpha)	777
19	1			*Methylocystis bryophila* (FN422003)	*Proteobacteria* (Alpha)	94.71	● *Proteobacteria* (Alpha)	699
20	1			*Uliginosibacterium gangwonense* (DQ665916)	*Proteobacteria* (Beta)	96.56	● *Proteobacteria* (Beta)	727
21	2			*Georgfuchsia toluolica* (EF219370)	*Proteobacteria* (Beta)	94.91	● *Proteobacteria* (Beta)	746
22	5			*Rhodoblastus sphagnicola* (AM040096)	*Proteobacteria* (Alpha)	98.93	● *Proteobacteria* (Alpha)	746
23	1			*Rhodoblastus sphagnicola* (AM040096)	*Proteobacteria* (Alpha)	99.12	● *Proteobacteria* (Alpha)	684
24	4			*Alkaliflexus imshenetskii* (AJ784993)	*Bacteroidetes*	87.82	● *Bacteroidetes*	737
25	2			*Alkaliflexus imshenetskii* (AJ784993)	*Bacteroidetes*	87.82	● *Bacteroidetes*	737
26	9	1		*Propionivibrio limicola* (AJ307983)	*Proteobacteria* (Beta)	95.49	● *Proteobacteria* (Beta)	732
27	1			*Methylocystis heyeri* (AM283543)	*Proteobacteria* (Alpha)	97.43	● *Proteobacteria* (Alpha)	779
28	1			*Melioribacter* roseus (CP003557)	*Chlorobi*	82.33	● Unclassified	765
29	1			*Telmatobacter bradus* (AM887760)	*Acidobacteria*	97.19	● *Acidobacteria*	747
30	1			*Acidicapsa ligni* (EU780204)	*Acidobacteria*	95.56	● *Acidobacteria*	675
31	2			*Telmatobacter bradus* (AM887760)	*Acidobacteria*	96.16	● *Acidobacteria*	756

Table S1. Phylogenetic affiliations of 16S rRNA gene clones (*Continued*)

Clone-phylotype No.	Total no. of clones			Related authentic species			Phylum (Class) classified by CLASSIFIER	Compared length (bp)
	Iris pseudacorus	*Scirpus juncoides*	Pond water	Specis (Accession no.)	Phylum (Class)	Identity (%)		
32	1			*Terrimicrobium sacchariphilum* (GU129926)	*Verrucomicrobia*	85.28	● *Verrucomicrobia*	673
33	1			*Skermanella aerolata* (DQ672568)	*Proteobacteria* (Alpha)	93.47	● *Proteobacteria* (Alpha)	704
34	1			*Fimbriimonas ginsengisoli* (GQ339893)	*Armatimonadetes*	88.38	● *Armatimonadetes*	749
35	1			*Sporotalea propionica* (AM258975)	*Firmicutes*	93.81	● *Firmicutes*	743
36	1			*Spirochaeta asiatica* (X93926)	*Spirochaetes*	86.85	● *Spirochaetes*	732
37	1			*Spirochaeta halophila* (M88722)	*Spirochaetes*	86.34	● *Spirochaetes*	776
38	2			*Haliangium tepidum* (AB062751)	*Proteobacteria* (Delta)	85.35	● *Proteobacteria* (Delta)	745
39	1			*Uliginosibacterium gangwonense* (DQ665916)	*Proteobacteria* (Beta)	99.13	● *Proteobacteria* (Beta)	808
40	1			*Dechloromonas agitata* (AF047462)	*Proteobacteria* (Beta)	98.27	● *Proteobacteria* (Beta)	750
41	1			*Akkermansia muciniphila* (CP001071)	*Verrucomicrobia*	83.14	● *Verrucomicrobia*	731
42	1			*Derxia gummosa* (AB089482)	*Proteobacteria* (Beta)	94.12	● *Proteobacteria* (Beta)	748
43	1			*Anaerosinus glycerini* (AJ010960)	*Firmicutes*	90.90	● *Firmicutes*	759
44	1			*Luteolibacter cuticulihirudinis* (JQ429496)	*Verrucomicrobia*	95.34	● *Verrucomicrobia*	772
45	1			*Brevifollis gellanilyticus* (AB552872)	*Verrucomicrobia*	82.73	● *Verrucomicrobia*	742
46	1			*Dictyoglomus turgidum* (CP001251)	*Chloroflexi*	81.85	● Unclassified	770
47	1			*Rhodoblastus sphagnicola*(AM040096)	*Proteobacteria* (Alpha)	98.52	● *Proteobacteria* (Alpha)	744
48	1			*Georgfuchsia toluolica* (EF219370)	*Proteobacteria* (Beta)	94.72	● *Proteobacteria* (Beta)	757
49	1			*Georgfuchsia toluolica* (EF219370)	*Proteobacteria* (Beta)	94.09	● *Proteobacteria* (Beta)	761
50	1			*Georgfuchsia toluolica* (EF219370)	*Proteobacteria* (Beta)	95.51	● *Proteobacteria* (Beta)	780
51	1	1		*Prosthecomicrobium hirschii*(HM037994)	*Proteobacteria* (Alpha)	94.49	● *Proteobacteria* (Alpha)	762
52	1		1	*Limnohabitans planktonicus* (FM165535)	*Proteobacteria* (Beta)	97.74	● *Proteobacteria* (Beta)	753
53	1	1	1	*Sediminibacterium goheungense* (JN674641)	*Bacteroidetes*	94.19	● *Bacteroidetes*	689
54	1	1		*Cystobacter miniatus* (DQ768111)	*Proteobacteria* (Delta)	94.78	● *Proteobacteria* (Delta)	672
55	1			*Methyloferula stellata* (ARWA01000001)	*Proteobacteria* (Alpha)	97.27	● *Proteobacteria* (Alpha)	697
56	1			*Microlunatus panaciterrae* (AB271051)	*Actinobacteria*	94.81	● *Actinobacteria*	696
57	1			*Aciditerrimonas ferrireducens* (AB517669)	*Actinobacteria*	89.21	● *Actinobacteria*	687
58	1			*Geothrix fermentans* (U41563)	*Acidobacteria*	94.35	● *Acidobacteria*	690
59	1			*Georgfuchsia toluolica* (EF219370)	*Proteobacteria* (Beta)	95.58	● *Proteobacteria* (Beta)	679
60	1			*Marinifilum fragile* (FJ394546)	*Bacteroidetes*	84.72	● *Bacteroidetes*	690
61	1			*Georgfuchsia toluolica* (EF219370)	*Proteobacteria* (Beta)	94.77	● *Proteobacteria* (Beta)	593
62	1			*Alkaliflexus imshenetskii* (AJ784993)	*Bacteroidetes*	87.43	● *Bacteroidetes*	682

Table S1. Phylogenetic affiliations of 16S rRNA gene clones (*Continued*)

Clone-phylotype No.	Total no. of clones			Related authentic species			Phylum (Class) classified by CLASSIFIER	Compared length (bp)
	Iris pseudacorus	*Scirpus juncoides*	Pond water	Specis (Accession no.)	Phylum (Class)	Identity (%)		
63	1			*Clostridium tertium* (Y18175)	*Firmicutes*	87.81	●*Firmicutes*	685
64	1			*Paludibaculum fermentans* (KJ461654)	*Acidobacteria*	90.94	●*Acidobacteria*	684
65	1			*Solitalea koreensis* (EU787448)	*Bacteroidetes*	82.02	●*Bacteroidetes*	685
66	1			*Telmatobacter bradus* (AM887760)	*Acidobacteria*	97.82	●*Acidobacteria*	687
67	1			*Methylocystis bryophila* (FN422003)	*Proteobacteria* (Alpha)	94.89	●*Proteobacteria* (Alpha)	685
68	1			*Phaeospirillum tilakii* (FN675262)	*Proteobacteria* (Alpha)	98.13	●*Proteobacteria* (Alpha)	644
69	2			*Spirochaeta asiatica* (X93926)	*Spirochaetes*	88.39	●*Spirochaetes*	690
70		1		*Pirellula staleyi* (CP001848)	*Planctomycetes*	86.25	●*Planctomycetes*	823
71		3		*Pseudanabaena tremula* (AF218371)	*Cyanobacteria*	90.93	●*Cyanobacteria*	748
72		1		*Geobacter sulfurreducens* subsp. *ethanolicus* (AB762695)	*Proteobacteria* (Delta)	77.78	●Unclassified	696
73		2		*Sphaerotilus natans* subsp. *sulfidivorans* (FJ871054)	*Proteobacteria* (Beta)	98.22	●*Proteobacteria* (Beta)	674
74		1		*Povalibacter uvarum* (AB548216)	*Proteobacteria* (Gamma)	91.26	●*Proteobacteria* (Gamma)	698
75		2		*Rubrivivax gelatinosus* (D16213)	*Proteobacteria* (Beta)	97.16	●*Proteobacteria* (Beta)	668
76		1		*Cupriavidus gilardii* (EU024163)	*Proteobacteria* (Beta)	90.10	●*Proteobacteria* (Beta)	828
77		1		*Ideonella azotifigens* (EU542576)	*Proteobacteria* (Beta)	98.28	●*Proteobacteria* (Beta)	820
78		1		*Ferriphaselus amnicola* (AB720115)	*Proteobacteria* (Beta)	93.63	●*Proteobacteria* (Beta)	738
79		1		*Chelativorans multitrophicus* (EF457243)	*Proteobacteria* (Alpha)	91.12	●*Proteobacteria* (Alpha)	743
80		2		*Hoeflea alexandrii* (AJ786600)	*Proteobacteria* (Alpha)	91.64	●*Proteobacteria* (Alpha)	801
81		6		*Ideonella dechloratans* (X72724)	*Proteobacteria* (Beta)	97.08	●*Proteobacteria* (Beta)	828
82		1		*Rubrivivax benzoatilyticus* (AEWG01000018)	*Proteobacteria* (Beta)	98.56	●*Proteobacteria* (Beta)	695
83		3		*Paludibaculum fermentans* (KJ461654)	*Acidobacteria*	91.64	●*Acidobacteria*	685
84		1		*Gemmata obscuriglobus* (ABGO01000325)	*Planctomycetes*	86.10	●*Planctomycetes*	705
85		1		*Paludibaculum fermentans* (KJ461654)	*Acidobacteria*	91.09	●*Acidobacteria*	694
86		1		*Aquincola tertiaricarbonis* (DQ656489)	*Proteobacteria* (Beta)	97.03	●*Proteobacteria* (Beta)	708
87		2		*Piscinibacter aquaticus* (DQ664244)	*Proteobacteria* (Beta)	99.69	●*Proteobacteria* (Beta)	643
88		1		*Piscinibacter aquaticus* (DQ664244)	*Proteobacteria* (Beta)	99.15	●*Proteobacteria* (Beta)	704
89		1		*Methylocystis bryophila* (FN422003)	*Proteobacteria* (Alpha)	94.56	●*Proteobacteria* (Alpha)	699
90		2		*Hyphomicrobium nitrativorans* (CP006912)	*Proteobacteria* (Alpha)	91.69	●*Proteobacteria* (Alpha)	710
91		1		*Paludibaculum fermentans* (KJ461654)	*Acidobacteria*	89.96	●*Acidobacteria*	737
92		1		*Ideonella azotifigens* (EU542576)	*Proteobacteria* (Beta)	98.34	●*Proteobacteria* (Beta)	728
93		1		*Lewinella cohaerens* (ARBR01000027)	*Bacteroidetes*	82.78	●*Bacteroidetes*	700

Table S1. Phylogenetic affiliations of 16S rRNA gene clones (*Continued*)

Clone-phylotype No.	Total no. of clones			Related authentic species			Phylum (Class) classified by CLASSIFIER	Compared length (bp)
	Iris pseudacorus	*Scirpus juncoides*	Pond water	Specis (Accession no.)	Phylum (Class)	Identity (%)		
94		1		*Terrimicrobium sacchariphilum* (GU129926)	*Verrucomicrobia*	90.61	● *Verrucomicrobia*	723
95		1		*Granulicella cerasi* (AB548308)	*Acidobacteria*	82.86	● *Acidobacteria*	829
96		1		*Bryobacter aggregatus* (AM162405)	*Acidobacteria*	90.58	● *Acidobacteria*	690
97		1		*Methyloferula stellata* (ARWA01000001)	*Proteobacteria* (Alpha)	97.10	● *Proteobacteria* (Delta)	689
98		1		*Coxiella burnetii* (CP000890)	*Proteobacteria* (Gamma)	89.90	● *Proteobacteria* (Gamma)	736
99		1		*Bradyrhizobium liaoningense* (AF208513)	*Proteobacteria* (Alpha)	99.57	● *Proteobacteria* (Delta)	690
100		1		*Bryobacter aggregatus* (AM162405)	*Acidobacteria*	90.81	● *Acidobacteria*	730
101		2		*Phormidium pristleyi* (AY493582)	*Cyanobacteria*	93.25	● *Cyanobacteria*	712
102		1		*Sandaracinus amylolyticus* (HQ540311)	*Proteobacteria* (Delta)	90.27	● *Proteobacteria* (Delta)	700
103		1		*Acidisphaera rubrifaciens* (D86512)	*Proteobacteria* (Alpha)	94.13	● *Proteobacteria* (Alpha)	716
104		1		*Paludibaculum fermentans* (KJ461654)	*Acidobacteria*	91.31	● *Acidobacteria*	702
105		2		*Aridibacter kavangonensis* (KF245633)	*Acidobacteria*	84.65	● *Acidobacteria*	711
106		1		*Bryobacter aggregatus* (AM162405)	*Acidobacteria*	90.86	● *Acidobacteria*	700
107		1		*Blastopirellula cremea* (JF748733)	*Planctomycetes*	85.61	● *Planctomycetes*	701
108		1		*Ilumatobacter fluminis* (AB360343)	*Actinobacteria*	89.53	● *Actinobacteria*	697
109		1		*Methyloversatilis thermotolerans* (KC782839)	*Proteobacteria* (Beta)	94.76	● *Proteobacteria* (Beta)	706
110		1		*Rubrivivax gelatinosus* (D16213)	*Proteobacteria* (Beta)	96.99	● *Proteobacteria* (Beta)	698
111		1		*Arthronema africanum* (AB115966)	*Cyanobacteria*	89.75	● *Cyanobacteria*	693
112		1		*Ignavibacterium album* (CP003418)	*Chlorobi*	83.90	● Unclassified	708
113		1		*Steroidobacter denitrificans* (EF605262)	*Proteobacteria* (Gamma)	91.19	● *Proteobacteria* (Gamma)	715
114		1		*Devosia albogilva* (EF433460)	*Proteobacteria* (Alpha)	91.32	● *Proteobacteria* (Alpha)	703
115		1		*Paucimonas lemoignei* (X92555)	*Proteobacteria* (Beta)	94.59	● *Proteobacteria* (Beta)	722
116		1		*Anaeromyxobacter dehalogenans* (AF382396)	*Proteobacteria* (Delta)	95.87	● *Proteobacteria* (Delta)	703
117		1		*Aciditerrimonas ferrireducens* (AB517669)	*Actinobacteria*	88.90	● *Actinobacteria*	695
118		2		*Paludibaculum fermentans* (KJ461654)	*Acidobacteria*	91.31	● *Acidobacteria*	679
119		1		*Skermanella aerolata* (DQ672568)	*Proteobacteria* (Alpha)	93.00	● *Proteobacteria* (Alpha)	700
120		1		*Aquincola tertiaricarbonis* (DQ656489)	*Proteobacteria* (Beta)	97.07	● *Proteobacteria* (Beta)	717
121		1		*Melittangium alboraceum* (AJ233907)	*Proteobacteria* (Delta)	93.73	● *Proteobacteria* (Delta)	686
122		1	1	*Chitinophaga ginsengisoli* (AB245374)	*Bacteroidetes*	87.57	● *Bacteroidetes*	694
123		1		*Gemmobacter tilapiae* (HQ111526)	*Proteobacteria* (Alpha)	97.66	● *Proteobacteria* (Alpha)	684
124		1		*Haliangium tepidum* (AB062751)	*Proteobacteria* (Delta)	89.60	● *Proteobacteria* (Delta)	693

Table S1. Phylogenetic affiliations of 16S rRNA gene clones (*Continued*)

Clone-phylotype No.	Total no. of clones			Related authentic species			Phylum (Class) classified by CLASSIFIER	Compared length (bp)
	Iris pseudacorus	*Scirpus juncoides*	Pond water	Specis (Accession no.)	Phylum (Class)	Identity (%)		
125		2		*Bryobacter aggregatus* (AM162405)	*Acidobacteria*	90.40	● *Acidobacteria*	677
126		1		*Bryobacter aggregatus* (AM162405)	*Acidobacteria*	91.73	● *Acidobacteria*	701
127		1		*Haliangium tepidum* (AB062751)	*Proteobacteria* (Delta)	90.25	● *Proteobacteria* (Delta)	680
128		1		*Armatimonas rosea* (AB529679)	*Armatimonadetes*	88.69	● *Armatimonadetes*	677
129		1		*Thauera mechernichensis* (Y17590)	*Proteobacteria* (Beta)	89.97	● *Proteobacteria* (Beta)	689
130		1		*Bradyrhizobium daqingense* (HQ231274)	*Proteobacteria* (Alpha)	99.71	● *Proteobacteria* (Alpha)	699
131		1		*Ignavibacterium album* (CP003418)	*Chlorobi*	84.66	● Unclassified	691
132		1		*Paludibaculum fermentans* (KJ461654)	*Acidobacteria*	92.30	● *Acidobacteria*	649
133		1		*Dongia mobilis* (FJ455532)	*Proteobacteria* (Alpha)	88.65	● *Proteobacteria* (Alpha)	696
134		1		*Terrimicrobium sacchariphilum* (GU129926)	*Verrucomicrobia*	83.95	● *Verrucomicrobia*	694
135		1		*Azoarcus buckelii* (AJ315676)	*Proteobacteria* (Beta)	93.00	● *Proteobacteria* (Beta)	686
136		1		*Nitrobacter vulgaris* (AM114522)	*Proteobacteria* (Alpha)	99.13	● *Proteobacteria* (Alpha)	686
137		1		*Aquicella siphonis* (AY359283)	*Proteobacteria* (Gamma)	92.43	● *Proteobacteria* (Gamma)	687
138		1		*Ilumatobacter fluminis* (AB360343)	*Actinobacteria*	91.69	● *Actinobacteria*	686
139		1		*Quadrisphaera granulorum* (AY831385)	*Actinobacteria*	96.63	● *Actinobacteria*	683
140		1		*Devosia albogilva* (EF433460)	*Proteobacteria* (Alpha)	96.10	● *Proteobacteria* (Alpha)	693
141		1		*Ideonella azotifigens* (EU542576)	*Proteobacteria* (Beta)	97.82	● *Proteobacteria* (Beta)	692
142		1		*Desulfovibrio butyratiphilus* (AB303305)	"Latescibacteria"	81.84	● "Latescibacteria"	685
143			4	*Flavobacterium macrobrachii* (FJ593904)	*Bacteroidetes*	97.12	● *Bacteroidetes*	764
144			1	*Formivibrio citricus* (Y17602)	*Proteobacteria* (Beta)	95.02	● *Proteobacteria* (Beta)	705
145			8	*Limnohabitans curvus* (AJ938026)	*Proteobacteria* (Beta)	97.42	● *Proteobacteria* (Beta)	776
146			2	*Limnohabitans parvus* (FM165536)	*Proteobacteria* (Beta)	97.36	● *Proteobacteria* (Beta)	759
147			1	*Limnohabitans curvus* (AJ938026)	*Proteobacteria* (Beta)	97.07	● *Proteobacteria* (Beta)	752
148			2	*Limnohabitans planktonicus* (FM165535)	*Proteobacteria* (Beta)	99.08	● *Proteobacteria* (Beta)	763
149			1	*Limnohabitans curvus* (AJ938026)	*Proteobacteria* (Beta)	97.71	● *Proteobacteria* (Beta)	743
150			1	*Curvibacter delicatus* (AF078756)	*Proteobacteria* (Beta)	97.50	● *Proteobacteria* (Beta)	760
151			10	*Limnohabitans curvus* (AJ938026)	*Proteobacteria* (Beta)	97.74	● *Proteobacteria* (Beta)	752
152			3	*Curvibacter delicatus* (AF078756)	*Proteobacteria* (Beta)	97.61	● *Proteobacteria* (Beta)	753
153			1	*Curvibacter delicatus* (AF078756)	*Proteobacteria* (Beta)	98.62	● *Proteobacteria* (Beta)	654
154			1	*Curvibacter delicatus* (AF078756)	*Proteobacteria* (Beta)	97.57	● *Proteobacteria* (Beta)	699
155			1	*Hydrogenophaga caeni* (DQ372983)	*Proteobacteria* (Beta)	96.37	● *Proteobacteria* (Beta)	689

Table S1. Phylogenetic affiliations of 16S rRNA gene clones (*Continued*)

Clone-phylotype No.	Total no. of clones			Related authentic species			Phylum (Class) classified by CLASSIFIER	Compared length (bp)
	Iris pseudacorus	*Scirpus juncoides*	Pond water	Specis (Accession no.)	Phylum (Class)	Identity (%)		
156			1	*Hydrogenophaga caeni* (DQ372983)	*Proteobacteria* (Beta)	96.37	*Proteobacteria* (Beta)	775
157			1	*Methylotenera mobilis* (CP001672)	*Proteobacteria* (Beta)	94.81	*Proteobacteria* (Beta)	693
158			1	*Methylophilus methylotrophus* (AB193724)	*Proteobacteria* (Beta)	99.60	*Proteobacteria* (Beta)	741
159			2	*Sediminibacterium goheungense* (JN674641)	*Bacteroidetes*	96.30	*Bacteroidetes*	703
160			2	*Sediminibacterium goheungense* (JN674641)	*Bacteroidetes*	95.92	*Bacteroidetes*	736
161			5	*Intrasporangium chromatireducens* (AWQS01000147)	*Actinobacteria*	89.84	*Actinobacteria*	738
162			2	*Cetobacterium somerae* (AJ438155)	*Fusobacteria*	99.22	*Fusobacteria*	771
163			1	*Dechloromonas hortensis* (AY277621)	*Proteobacteria* (Beta)	97.55	*Proteobacteria* (Beta)	736
164			1	*Phenylobacterium composti* (EU022524)	*Proteobacteria* (Alpha)	87.32	*Proteobacteria* (Alpha)	703
165			2	*Pseudarcicella hirudinis* (HE585218)	*Bacteroidetes*	90.96	*Bacteroidetes*	697
166			1	*Solitalea canadensis* (AGSC01000046)	*Bacteroidetes*	85.34	*Bacteroidetes*	771
167			1	*Haliscomenobacter calcifugiens* (AJ786327)	*Bacteroidetes*	85.57	*Bacteroidetes*	673
168			1	*Luteibaculum oceani* (KC169812)	*Bacteroidetes*	86.29	*Bacteroidetes*	715
169			1	*Tabrizicola aquatica* (HQ392507)	*Proteobacteria* (Alpha)	97.82	*Proteobacteria* (Alpha)	733
170			1	*Ferruginibacter alkalilentus* (FJ263934)	*Bacteroidetes*	94.92	*Bacteroidetes*	729
171			1	*Tabrizicola aquatica* (HQ392507)	*Proteobacteria* (Alpha)	97.94	*Proteobacteria* (Alpha)	679
172			3	*Pseudarcicella hirudinis* (HE585218)	*Bacteroidetes*	91.33	*Bacteroidetes*	750
173			1	*Microcystis ichthyoblabe* (AB035550)	*Cyanobacteria*	98.96	*Cyanobacteria*	670
174			2	*Polynucleobacter necessarius* subsp. *asymbioticus* (CP000655)	*Proteobacteria* (Beta)	99.86	*Proteobacteria* (Beta)	738
175			1	*Pseudarcicella hirudinis* (HE585218)	*Bacteroidetes*	90.95	*Bacteroidetes*	696
176			1	*Limnohabitans planktonicus* (FM165535)	*Proteobacteria* (Beta)	94.47	*Proteobacteria* (Beta)	705
177			1	*Polynucleobacter necessarius* subsp. *asymbioticus* (CP000655)	*Proteobacteria* (Beta)	99.56	*Proteobacteria* (Beta)	676
178			1	*Polynucleobacter necessarius* subsp. *asymbioticus* (CP000655)	*Proteobacteria* (Beta)	100	*Proteobacteria* (Beta)	677
179			1	*Thioprofundum lithotrophicum* (AB468957)	*Proteobacteria* (Gamma)	93.84	*Proteobacteria* (Gamma)	699
180			1	*Sediminibacterium goheungense* (JN674641)	*Bacteroidetes*	96.04	*Bacteroidetes*	733
181			1	*Gloeobacter violaceus* (BA000045)	*Cyanobacteria*	82.60	*Cyanobacteria*	762
182			1	*Methylotenera mobilis* (DQ287786)	*Proteobacteria* (Beta)	98.24	*Proteobacteria* (Beta)	626
183			1	*Desulfonatronum lacustre* (AF418171)	*Proteobacteria* (Delta)	80.35	*Proteobacteria* (Unclassified)	684
184			1	*Haematobacter massiliensis* (DQ342309)	*Proteobacteria* (Alpha)	98.26	*Proteobacteria* (Alpha)	689
185			1	*Calothrix desertica* (AF132779)	*Cyanobacteria*	98.17	*Cyanobacteria*	765
186			1	*Mesoflavibacter sabulilitoris* (KJ816860)	*Bacteroidetes*	78.55	*Bacteroidetes*	724

Table S1. Phylogenetic affiliations of 16S rRNA gene clones (*Continued*)

Clone-phylotype No.	Total no. of clones			Related authentic species			Phylum (Class) classified by CLASSIFIER	Compared length (bp)
	Iris pseudacorus	*Scirpus juncoides*	Pond water	Specis (Accession no.)	Phylum (Class)	Identity (%)		
187			1	*Fimbriimonas ginsengisoli* (CP002763)	*Armatimonadetes*	88.75	● *Armatimonadetes*	712
188			1	*Cryobacterium psychrotolerans* (DQ515963)	*Actinobacteria*	94.99	● *Actinobacteria*	678
189			1	*Desulforegula conservatrix* (AUEY01000125)	*Proteobacteria* (Delta)	84.13	● *Proteobacteria* (Delta)	694
190			1	Methylobacter psychrophilus (AF152597)	*Proteobacteria* (Gamma)	94.24	● *Proteobacteria* (Gamma)	678
191			1	*Kaistia defluvii* (AM409365)	*Proteobacteria* (Alpha)	85.91	● *Proteobacteria* (Alpha)	655
192			1	*Aggregicoccus edonensis* (KF767690)	*Proteobacteria* (Delta)	86.29	● *Proteobacteria* (Delta)	672
193			1	*Flavobacterium cheonanense* (GU295968)	*Bacteroidetes*	96.78	● *Bacteroidetes*	683
194			1	*Novosphingobium lentum* (AJ303009)	*Proteobacteria* (Alpha)	94.80	● *Proteobacteria* (Alpha)	714
195			1	*Streptomyces regensis* (DQ026649)	*Actinobacteria*	90.13	● *Actinobacteria*	679
196			2	*Alterococcus agarolyticus* (AF075271)	*Verrucomicrobia*	84.99	● *Verrucomicrobia*	673
197			1	*Limnohabitans parvus* (FM165536)	*Proteobacteria* (Beta)	97.34	● *Proteobacteria* (Beta)	676
198			2	*Aeromonas tecta* (AJ458402)	*Proteobacteria* (Gamma)	100	● *Proteobacteria* (Gamma)	636
	95	95	95					

Partial sequences of the clones shown in bold type were determined, and applied to phylogenetic analysis

The color of circle symbol in the column of "Phylum (Class) classified by CLASSIFIER" corresponds to that of bar describing each phylum in Fig. S1

Table S2. Isolated microbes in this study and their related authentic species on the basis of 16S rRNA gene sequence

Isolate-phylotype No.	Total no. of isolates										Related authentic species			Phylum (Class) classified by CLASSIFIER	Compared length (bp)
	Iris pseudacorus				*Scirpus juncoides*				Pond water						
	DTSA	DTSG	DR2A	DR2G	DTSA	DTSG	DR2A	DR2G	DTSA	DTSG	Species (Accession no.)	Phylum (Class)	Identity (%)		
1	2	4	1	1							*Rhizomicrobium electricum* (AB365487)	*Proteobacteria* (Alpha)	96.02	●*Proteobacteria* (Alpha)	728
2	1	2									*Rhizomicrobium palustre* (AB081581)	*Proteobacteria* (Alpha)	97.96	●*Proteobacteria* (Alpha)	736
3	1										*Novosphingobium fuchskuhlense* (JN399172)	*Proteobacteria* (Alpha)	99.58	●*Proteobacteria* (Alpha)	721
4	3	1		1							*Aminobacter anthyllidis* (FR869633)	*Proteobacteria* (Alpha)	91.44	●*Proteobacteria* (Alpha)	794
5	2	2									*Roseiarcus fermentans* (KJ406703)	*Proteobacteria* (Alpha)	96.88	●*Proteobacteria* (Alpha)	738
6	2	1									*Paludibacterium yongneupense* (AM396358)	*Proteobacteria* (Beta)	97.83	●*Proteobacteria* (Beta)	739
7	1	1		1							*Paludibacterium yongneupense* (AM396358)	*Proteobacteria* (Beta)	97.41	●*Proteobacteria* (Beta)	734
8	1										*Rhodoblastus acidophilus* (FR733696)	*Proteobacteria* (Alpha)	99.31	●*Proteobacteria* (Alpha)	722
9	1	1	1	1							*Rhodoblastus acidophilus* (FR733696)	*Proteobacteria* (Alpha)	99.17	●*Proteobacteria* (Alpha)	719
10	1										*Novosphingobium sediminicola* (FJ177534)	*Proteobacteria* (Alpha)	98.56	●*Proteobacteria* (Alpha)	709
11	2	1									*Rhodopseudomonas thermotolerans* (FR851928)	*Proteobacteria* (Alpha)	99.86	●*Proteobacteria* (Alpha)	705
12	1	1									*Bradyrhizobium iriomotense* (X87272)	*Proteobacteria* (Alpha)	100	●*Proteobacteria* (Alpha)	715
13	1										*Herbaspirillum aquaticum* (FJ267649)	*Proteobacteria* (Beta)	100	●*Proteobacteria* (Beta)	744
14	1										*Opitutus terrae* (AJ229235)	*Verrucomicrobia*	89.50	●*Verrucomicrobia*	734
15	3	2									*Telmatobacter bradus* (AM887760)	*Acidobacteria*	97.16	●*Acidobacteria*	740
16	2										*Rhizomicrobium palustre* (AB081581)	*Proteobacteria* (Alpha)	97.20	●*Proteobacteria* (Alpha)	642
17	1	2	1								*Methylocystis parvus* (AJTV01000076)	*Proteobacteria* (Alpha)	92.56	●*Proteobacteria* (Alpha)	739
18	1										*Roseiarcus fermentans* (KJ406703)	*Proteobacteria* (Alpha)	98.07	●*Proteobacteria* (Alpha)	710
19	1										*Ideonella dechloratans* (X72724)	*Proteobacteria* (Beta)	98.56	●*Proteobacteria* (Beta)	696
20	1										*Mycobacterium moriokaense* (AJ429044)	*Actinobacteria*	99.86	●*Actinobacteria*	708
21	3		1								*Rhodoblastus sphagnicola* (AM040096)	*Proteobacteria* (Alpha)	99.17	●*Proteobacteria* (Alpha)	719
22	1										*Telmatospirillum siberiense* (AF524863)	*Proteobacteria* (Alpha)	97.79	●*Proteobacteria* (Alpha)	723
23	3	4	2	3							*Uliginosibacterium gangwonense* (DQ665916)	*Proteobacteria* (Beta)	96.60	●*Proteobacteria* (Beta)	735
24	5										*Bradyrhizobium elkanii* (U35000)	*Proteobacteria* (Alpha)	99.44	●*Proteobacteria* (Alpha)	714
25	2	3		2							*Uliginosibacterium gangwonense* (DQ665916)	*Proteobacteria* (Beta)	96.64	●*Proteobacteria* (Beta)	715
26	1										*Flavitalea populi* (HM130561)	*Bacteroidetes*	93.46	●*Bacteroidetes*	703
27	1			1							*Rhizomicrobium electricum* (AB365487)	*Proteobacteria* (Alpha)	95.83	●*Proteobacteria* (Alpha)	695
28		3	1								*Ideonella dechloratans* (X72724)	*Proteobacteria* (Beta)	98.75	●*Proteobacteria* (Beta)	725
29		1		3							*Telmatobacter bradus* (AM887760)	*Acidobacteria*	97.03	●*Acidobacteria*	740
30		1									*Telmatobacter bradus* (AM887760)	*Acidobacteria*	95.39	●*Acidobacteria*	738
31		1									*Acidobacterium capsulatum* (CP001472)	*Acidobacteria*	91.88	●*Acidobacteria*	739
32		2									*Rubrivivax benzoatilyticus* (AEWG01000018)	*Proteobacteria* (Beta)	97.68	●*Proteobacteria* (Beta)	732
33		1									*Uliginosibacterium gangwonense* (DQ665916)	*Proteobacteria* (Beta)	99.20	●*Proteobacteria* (Beta)	747
34		1									*Opitutus terrae* (CP001032)	*Verrucomicrobia*	92.88	●*Verrucomicrobia*	702
35		1					1				*Tepidamorphus gemmatus* (GU187912)	*Proteobacteria* (Alpha)	91.19	●*Proteobacteria* (Alpha)	715
36		1									*Flavitalea populi* (HM130561)	*Bacteroidetes*	93.64	●*Bacteroidetes*	723
37		1									*Propionicimonas paludicola* (AB078858)	*Actinobacteria*	96.92	●*Actinobacteria*	714

Table S2. Isolated microbes in this study and their related authentic species on the basis of 16S rRNA gene sequence (*Continued*)

Isolate-phylotype No.	Total no. of isolates										Related authentic species			Phylum (Class) classified by CLASSIFIER	Compared length (bp)
	Iris pseudacorus				*Scirpus juncoides*				Pond water						
	DTSA	DTSG	DR2A	DR2G	DTSA	DTSG	DR2A	DR2G	DTSA	DTSG	Species (Accession no.)	Phylum (Class)	Identity (%)		
38		1									*Opitutus terrae* (AJ229235)	*Verrucomicrobia*	94.90	● *Verrucomicrobia*	725
39		1									*Novosphingobium lentum* (AJ303009)	*Proteobacteria* (Alpha)	98.30	● *Proteobacteria* (Alpha)	717
40		1									*Novosphingobium lentum* (AB682668)	*Proteobacteria* (Alpha)	98.00	● *Proteobacteria* (Alpha)	705
41		3	2	1							*Bradyrhizobium denitrificans* (X66025)	*Proteobacteria* (Alpha)	100	● *Proteobacteria* (Alpha)	695
42		1									*Methylocystis heyeri* (AM283543)	*Proteobacteria* (Alpha)	96.57	● *Proteobacteria* (Alpha)	700
43			2								*Kineosporia aurantiaca* (AB003931)	*Actinobacteria*	95.44	● *Actinobacteria*	818
44			1	1							*Azospirillum halopraeferens* (Z29618)	*Proteobacteria* (Alpha)	93.80	● *Proteobacteria* (Alpha)	786
45			2								*Telmatobacter bradus* (AM887760)	*Acidobacteria*	96.15	● *Acidobacteria*	808
46			1								*Rubrivivax gelatinosus* (D16213)	*Proteobacteria* (Beta)	96.93	● *Proteobacteria* (Beta)	814
47			1								*Alsobacter metallidurans* (AB231946)	*Proteobacteria* (Alpha)	97.10	● *Proteobacteria* (Alpha)	796
48			1	2							*Bradyrhizobium huanghuaihaiense* (HQ231463)	*Proteobacteria* (Alpha)	100	● *Proteobacteria* (Alpha)	789
49			3								*Bradyrhizobium daqingense* (HQ231274)	*Proteobacteria* (Alpha)	99.30	● *Proteobacteria* (Alpha)	788
50			1								*Geothrix fermentans* (U41563)	*Acidobacteria*	97.27	● *Acidobacteria*	807
51			2								*Novosphingobium ginsenosidimutans* (JQ349046)	*Proteobacteria* (Alpha)	97.85	● *Proteobacteria* (Alpha)	789
52			1								*Paludibaculum fermentans* (KJ461654)	*Acidobacteria*	93.54	● *Acidobacteria*	666
53			1								*Opitutus terrae* (AJ229235)	*Verrucomicrobia*	94.26	● *Verrucomicrobia*	662
54				1							*Telmatospirillum siberiense* (AF524863)	*Proteobacteria* (Alpha)	97.83	● *Proteobacteria* (Alpha)	736
55				1							*Derxia gummosa* (AB089482)	*Proteobacteria* (Beta)	94.35	● *Proteobacteria* (Beta)	779
56				1							*Telmatospirillum siberiense* (AF524863)	*Proteobacteria* (Alpha)	94.14	● *Proteobacteria* (Alpha)	787
57				1							*Rhizomicrobium electricum* (AB365487)	*Proteobacteria* (Alpha)	96.26	● *Proteobacteria* (Alpha)	776
59				1							*Skermanella aerolata* (NR_043929)	*Proteobacteria* (Alpha)	92.58	● *Proteobacteria* (Alpha)	788
60				1							*Rhizomicrobium palustre* (AB081581)	*Proteobacteria* (Alpha)	97.46	● *Proteobacteria* (Alpha)	788
61				1							*Carboxylicivirga mesophila* (JQ672625)	*Bacteroidetes*	87.73	● *Bacteroidetes*	785
62					3	2	1	4			*Bradyrhizobium oligotrophica* (AP012603)	*Proteobacteria* (Alpha)	99.85	● *Proteobacteria* (Alpha)	666
63					2	2					*Roseateles aquatilis* (AM501446)	*Proteobacteria* (Beta)	97.60	● *Proteobacteria* (Beta)	667
64					1	1					*Rhizomicrobium palustre* (AB081581)	*Proteobacteria* (Alpha)	95.28	● *Proteobacteria* (Alpha)	720
65					1						*Rubrivivax gelatinosus* (D16213)	*Proteobacteria* (Beta)	98.36	● *Proteobacteria* (Beta)	670
66					1						*Rubrivivax gelatinosus* (D16213)	*Proteobacteria* (Beta)	97.58	● *Proteobacteria* (Beta)	661
67					2			1			*Aquabacterium commune* (NR_024875)	*Proteobacteria* (Beta)	96.35	● *Proteobacteria* (Beta)	712
68					1	2		3			*Devosia albogilva* (NR_044212)	*Proteobacteria* (Alpha)	91.88	● *Proteobacteria* (Alpha)	677
69					1						*Hyphomicrobium nitrativorans* (CP006912)	*Proteobacteria* (Alpha)	91.95	● *Proteobacteria* (Alpha)	671
70					4	3					*Piscinibacter aquaticus* (NR_043921)	*Proteobacteria* (Beta)	99.04	● *Proteobacteria* (Beta)	833
71					1	1					*Caulobacter fusiformis* (AJ227759)	*Proteobacteria* (Alpha)	95.77	● *Proteobacteria* (Alpha)	709
72					1						*Ensifer adhaerens* (AM181733)	*Proteobacteria* (Alpha)	97.65	● *Proteobacteria* (Alpha)	723
73					1						*Opitutus terrae* (AJ229235)	*Verrucomicrobia*	94.71	● *Verrucomicrobia*	719
74					1						*Janthinobacterium agaricidamnosum* (HQ699437)	*Proteobacteria* (Alpha)	95.74	● *Proteobacteria* (Alpha)	727
75					1			2			*Prosthecomicrobium hirschii* (HM037994)	*Proteobacteria* (Alpha)	93.01	● *Proteobacteria* (Alpha)	701

Table S2. Isolated microbes in this study and their related authentic species on the basis of 16S rRNA gene sequence (*Continued*)

Isolate-phylotype No.	Total no. of isolates										Related authentic species			Phylum (Class) classified by CLASSIFIER	Compared length (bp)
	Iris pseudacorus				*Scirpus juncoides*				Pond water						
	DTSA	DTSG	DR2A	DR2G	DTSA	DTSG	DR2A	DR2G	DTSA	DTSG	Species (Accession no.)	Phylum (Class)	Identity (%)		
76					2						*Curvibacter delicatus* (AF078756)	*Proteobacteria* (Alpha)	98.62	● *Proteobacteria* (Alpha)	725
77					1						*Hyphomicrobium facile* subsp. *tolerans* (Y14311)	*Proteobacteria* (Alpha)	99.38	● *Proteobacteria* (Alpha)	648
78					1	1					*Methylibium petroleiphilum* (AF176594)	*Proteobacteria* (Beta)	98.18	● *Proteobacteria* (Beta)	715
79					1						*Rhizomicrobium palustre* (AB081581)	*Proteobacteria* (Alpha)	98.47	● *Proteobacteria* (Alpha)	718
80					1						*Telmatobacter bradus* (AM887760)	*Acidobacteria*	96.07	● *Acidobacteria*	712
81					4	4					*Rubrivivax gelatinosus* (D16213)	*Proteobacteria* (Beta)	97.50	● *Proteobacteria* (Beta)	720
82					2						*Bradyrhizobium lablabi* (GU433448)	*Proteobacteria* (Alpha)	99.56	● *Proteobacteria* (Alpha)	685
83					2						*Roseiarcus fermentans* (KJ406703)	*Proteobacteria* (Alpha)	97.43	● *Proteobacteria* (Alpha)	622
84					1						*Kineosporia rhamnosa* (AB003935)	*Actinobacteria*	96.54	● *Actinobacteria*	668
85					1						*Phenylobacterium lituiforme* (AY534887)	*Proteobacteria* (Alpha)	96.71	● *Proteobacteria* (Alpha)	668
86					1						*Azoarcus buckelii* (AJ315676)	*Proteobacteria* (Beta)	95.48	● *Proteobacteria* (Beta)	664
87					7	6	5				*Ideonella azotifigens* (EU542576)	*Proteobacteria* (Beta)	98.34	● *Proteobacteria* (Beta)	725
88						4		2			*Devosia yakushimensis* (AB682141)	*Proteobacteria* (Alpha)	91.18	● *Proteobacteria* (Alpha)	795
89						2					*Ideonella dechloratans* (X72724)	*Proteobacteria* (Beta)	98.75	● *Proteobacteria* (Beta)	724
90						1					*Rhodoblastus acidophilus* (FR733696)	*Proteobacteria* (Alpha)	98.35	● *Proteobacteria* (Alpha)	787
91						2					*Bradyrhizobium japonicum* (AP012206)	*Proteobacteria* (Alpha)	99.35	● *Proteobacteria* (Alpha)	768
92						1					*Sinorhizobium chiapanecum* (EU286550)	*Proteobacteria* (Alpha)	93.24	● *Proteobacteria* (Alpha)	725
93						1					*Rhizomicrobium palustre* (AB081581)	*Proteobacteria* (Alpha)	98.55	● *Proteobacteria* (Alpha)	761
94						1					*Telmatobacter bradus* (AM887760)	*Acidobacteria*	96.56	● *Acidobacteria*	788
95						2					*Opitutus terrae* (AJ229235)	*Verrucomicrobia*	94.77	● *Verrucomicrobia*	727
96						1					*Kineococcus xinjiangensis* (JQ314347)	*Actinobacteria*	93.46	● *Actinobacteria*	797
97						1					*Terrimonas lutea* (AB192292)	*Bacteroidetes*	91.70	● *Bacteroidetes*	723
98						1					*Rhodobium orientis* (D30792)	*Proteobacteria* (Alpha)	89.86	● *Proteobacteria* (Alpha)	732
99						1					*Azoarcus buckelii* (AJ315676)	*Proteobacteria* (Beta)	87.82	● *Proteobacteria* (Beta)	790
100						1					*Rhodobacter blasticus* (DQ342322)	*Proteobacteria* (Alpha)	97.12	● *Proteobacteria* (Alpha)	729
101						1					*Opitutus terrae* (AJ229235)	*Verrucomicrobia*	94.06	● *Verrucomicrobia*	724
102						1					*Acidicapsa ligni* (EU780204)	*Acidobacteria*	96.31	● *Acidobacteria*	732
103						1					*Sporichthya polymorpha* (AB025317)	*Actinobacteria*	93.95	● *Actinobacteria*	777
104						1					*Roseiarcus fermentans* (KJ406703)	*Proteobacteria* (Alpha)	96.75	● *Proteobacteria* (Alpha)	707
105							1				*Ideonella dechloratans* (X72724)	*Proteobacteria* (Beta)	96.42	● *Proteobacteria* (Beta)	816
106							1				*Bradyrhizobium daqingense* (HQ231274)	*Proteobacteria* (Alpha)	99.29	● *Proteobacteria* (Alpha)	790
107							1	1			*Aquincola tertiaricarbonis* (DQ656489)	*Proteobacteria* (Beta)	97.24	● *Proteobacteria* (Beta)	799
108							1				*Novosphingobium ginsenosidimutans* (JQ349046)	*Proteobacteria* (Alpha)	98.36	● *Proteobacteria* (Alpha)	791
109							1				*Steroidobacter denitrificans* (EF605262)	*Proteobacteria* (Gamma)	88.12	● *Proteobacteria* (Gamma)	827
110							2				*Roseiarcus fermentans* (KJ406703)	*Proteobacteria* (Alpha)	95.91	● *Proteobacteria* (Alpha)	811
111							1				*Tepidamorphus gemmatus* (GU187912)	*Proteobacteria* (Alpha)	91.03	● *Proteobacteria* (Alpha)	818
112							1				*Prosthecomicrobium hirschii* (GQ221763)	*Proteobacteria* (Alpha)	94.42	● *Proteobacteria* (Alpha)	812

Table S2. Isolated microbes in this study and their related authentic species on the basis of 16S rRNA gene sequence (*Continued*)

Isolate-phylotype No.	Total no. of isolates										Related authentic species			Phylum (Class) classified by CLASSIFIER	Compared length (bp)
	Iris pseudacorus				*Scirpus juncoides*				Pond water						
	DTSA	DTSG	DR2A	DR2G	DTSA	DTSG	DR2A	DR2G	DTSA	DTSG	Species (Accession no.)	Phylum (Class)	Identity (%)		
113							1				*Methylibium petroleiphilum* (AF176594)	*Proteobacteria* (Beta)	97.32	●*Proteobacteria* (Beta)	821
114							1				*Rhodoblastus sphagnicola* (AM040096)	*Proteobacteria* (Alpha)	98.61	●*Proteobacteria* (Alpha)	792
115							1				*Roseomonas aquatica* (AM231587)	*Proteobacteria* (Alpha)	93.34	●*Proteobacteria* (Alpha)	796
116							1				*Ancylobacter defluvii* (KC243678)	*Proteobacteria* (Alpha)	93.66	●*Proteobacteria* (Alpha)	813
117							1				*Opitutus terrae* (AJ229235)	*Verrucomicrobia*	91.89	●*Verrucomicrobia*	838
118							1				*Labrys okinawensis* (AB236169)	*Proteobacteria* (Alpha)	93.30	●*Proteobacteria* (Alpha)	810
119							1				*Hyphomicrobium hollandicum* (Y14303)	*Proteobacteria* (Alpha)	90.96	●*Proteobacteria* (Alpha)	816
120							1				*Telmatospirillum siberiense* (NR_041925)	*Proteobacteria* (Alpha)	95.05	●*Proteobacteria* (Alpha)	813
121							1				*Beijerinckia mobilis* (AJ563932)	*Proteobacteria* (Alpha)	93.28	●*Proteobacteria* (Alpha)	791
122								4			*Ideonella dechloratans* (X72724)	*Proteobacteria* (Beta)	98.87	●*Proteobacteria* (Beta)	798
123								1			*Caulobacter profunda* (KF360052)	*Proteobacteria* (Alpha)	95.84	●*Proteobacteria* (Alpha)	789
124								4			*Aquabacterium commune* (AF035054)	*Proteobacteria* (Beta)	97.20	●*Proteobacteria* (Beta)	787
125								1			*Asticcacaulis excentricus* (AB016610)	*Proteobacteria* (Alpha)	95.84	●*Proteobacteria* (Alpha)	721
126								1			*Prosthecomicrobium hirschii* (HM037994)	*Proteobacteria* (Alpha)	95.53	●*Proteobacteria* (Alpha)	788
127								1			*Hyphomicrobium hollandicum* (Y14303)	*Proteobacteria* (Alpha)	90.86	●*Proteobacteria* (Alpha)	789
128									2	11	*Novosphingobium fuchskuhlense* (AF235996)	*Proteobacteria* (Alpha)	99.44	●*Proteobacteria* (Alpha)	710
129									1	1	*Herbaspirillum seropedicae* (Y10146)	*Proteobacteria* (Beta)	97.88	●*Proteobacteria* (Beta)	708
130									10		*Pelomonas puraquae* (AM501439)	*Proteobacteria* (Beta)	99.72	●*Proteobacteria* (Beta)	714
131									22		*Limnohabitans curvus* (AJ938026)	*Proteobacteria* (Beta)	97.90	●*Proteobacteria* (Beta)	713
132									1	18	*Flavobacterium cheonanense* (GU295968)	*Bacteroidetes*	97.24	●*Bacteroidetes*	726
133									2		*Novosphingobium ginsenosidimutans* (JQ349046)	*Proteobacteria* (Alpha)	99.58	●*Proteobacteria* (Alpha)	720
134									1		*Novosphingobium naphthalenivorans* (AB177883)	*Proteobacteria* (Alpha)	94.80	●*Proteobacteria* (Alpha)	712
135									2	3	*Sandarakinorhabdus limnophila* (AY902680)	*Proteobacteria* (Alpha)	99.16	●*Proteobacteria* (Alpha)	714
136									2		*Intrasporangium mesophilum* (FJ428831)	*Actinobacteria*	90.47	●*Actinobacteria*	703
137									2	1	*Tabrizicola aquatica* (HQ392507)	*Proteobacteria* (Alpha)	98.11	●*Proteobacteria* (Alpha)	689
138									0	1	*Flavobacterium aquidurense* (JX657043)	*Bacteroidetes*	95.75	●*Bacteroidetes*	777
139									0	1	*Flavobacterium fontis* (JN873147)	*Bacteroidetes*	98.14	●*Bacteroidetes*	808
140									0	3	*Aeromonas tecta* (AJ458402)	*Proteobacteria* (Gamma)	99.51	●*Proteobacteria* (Gamma)	818
141									0	1	*Aeromonas sharmana* (DQ013306)	*Proteobacteria* (Gamma)	96.18	●*Proteobacteria* (Gamma)	813
142									0	3	*Flavobacterium koreense* (GU295967)	*Bacteroidetes*	96.67	●*Bacteroidetes*	814
143									0	1	*Taibaiella koreensis* (KC252613)	*Bacteroidetes*	90.09	●*Bacteroidetes*	777
144									0	1	*Hyphomicrobium nitrativorans* (CP006912)	*Proteobacteria* (Alpha)	92.73	●*Proteobacteria* (Alpha)	772
Total	45	45	25	25	45	45	25	25	45	45					

Partial sequences of the strains shown in bold type were determined, and applied to phylogenetic analysis

The color of circle symbol in the column of "Phylum (Class) classified by CLASSIFIER" corresponds to that of bar describing each phylum in Fig. S1

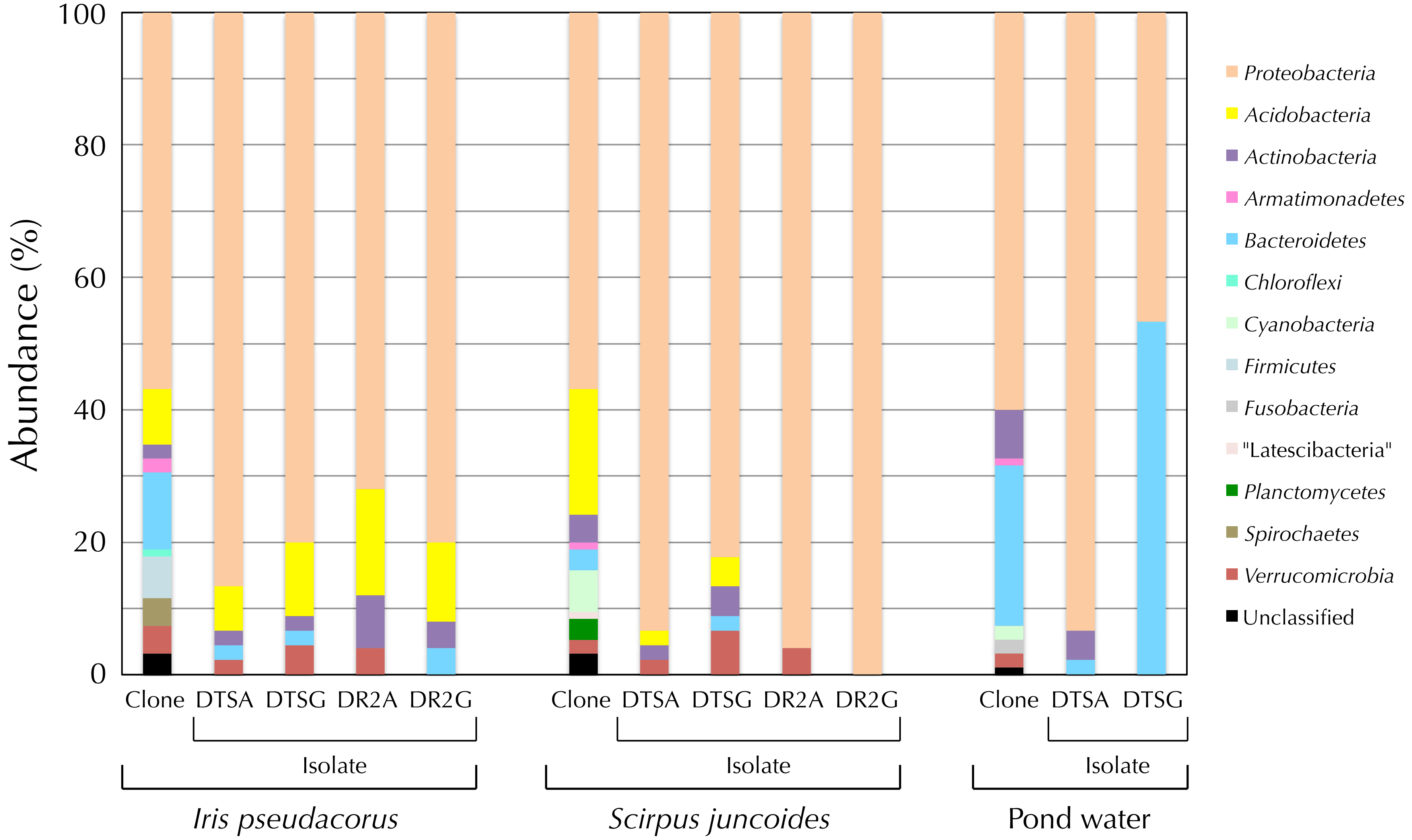

Fig. S1
Tanaka *et al.*

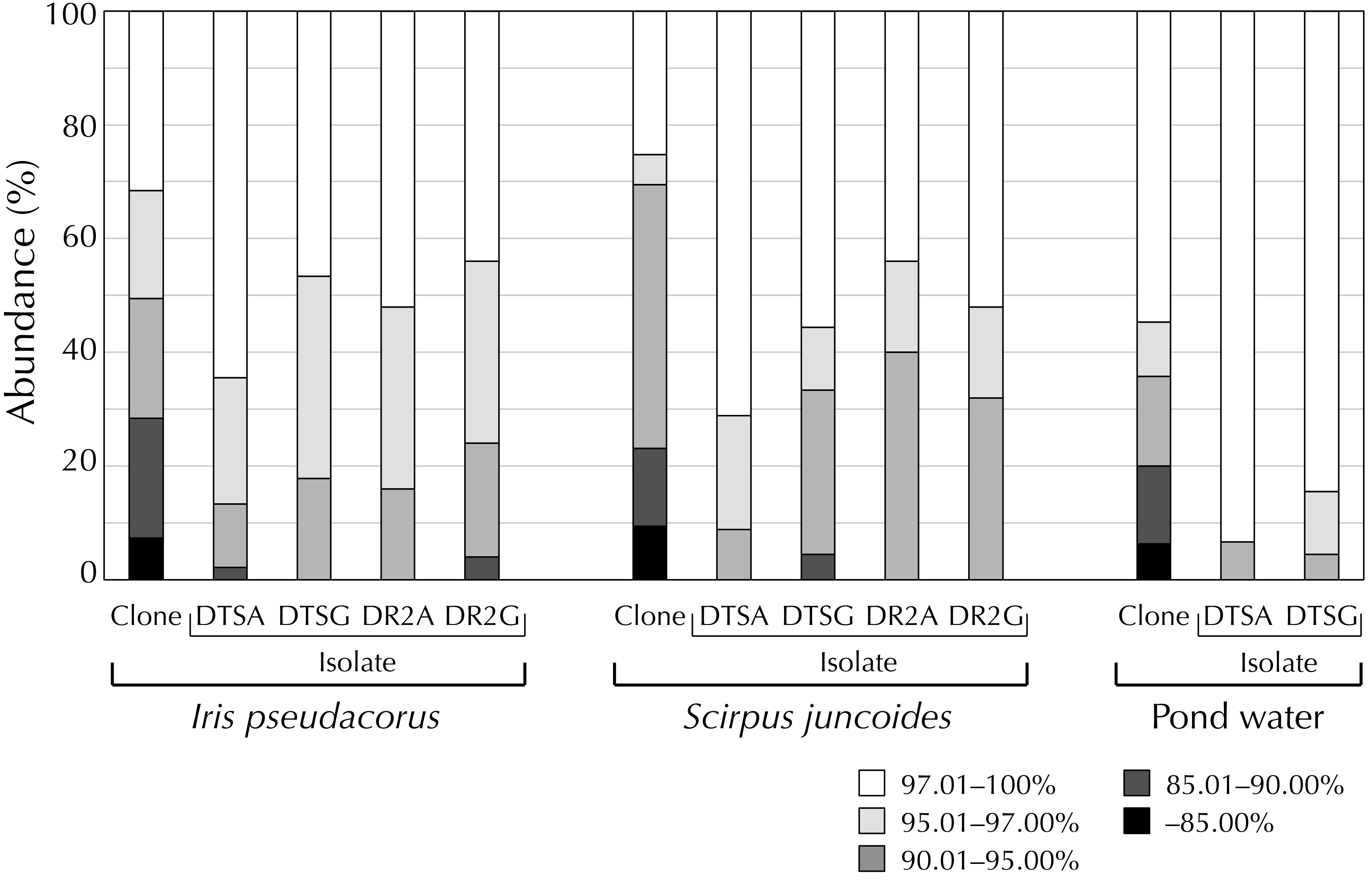

Fig. S2
Tanaka *et al.*

Figure legends

Fig. S1. Phylogenetic distribution of the 16S rRNA gene clones and isolates at the level of phylum in the roots of two emergent plants, *I. pseudacorus* and *S. juncoides*, and pond water.

Fig. S2. Abundance of novel clonal 16S rRNA gene sequences and isolates obtained from the roots of two emergent plants, *I. pseudacorus* and *S. juncoides*, and pond water. The similarity percentages between the clones or isolates and their closest authentic species in the EZ BioCloud database are shown.
